# Epstein-Barr Virus Latent Membrane Protein 1 Regulates Host B Cell MicroRNA-155 and Its Target FOXO3a *via* PI3K p110α Activation

**DOI:** 10.3389/fmicb.2019.02692

**Published:** 2019-11-26

**Authors:** Olivia Hatton, Madeline M. Smith, Madison Alexander, Melanie Mandell, Carissa Sherman, Madeline W. Stesney, Sin Ting Hui, Gillian Dohrn, Joselinne Medrano, Kurt Ringwalt, Aleishia Harris-Arnold, Eden M. Maloney, Sheri M. Krams, Olivia M. Martinez

**Affiliations:** ^1^Department of Molecular Biology, Colorado College, Colorado Springs, CO, United States; ^2^Division of Abdominal Transplantation, Department of Surgery, Stanford University School of Medicine, Stanford, CA, United States; ^3^Stanford Immunology, Stanford University School of Medicine, Stanford, CA, United States

**Keywords:** Epstein-Barr virus, latent membrane protein 1, microRNA, miR-155, FOXO3a, PI3K

## Abstract

Epstein-Barr Virus (EBV) is associated with potentially fatal lymphoproliferations such as post-transplant lymphoproliferative disorder (PTLD), a serious complication of transplantation. The viral mechanisms underlying the development and maintenance of EBV+ B cell lymphomas remain elusive but represent attractive therapeutic targets. EBV modulates the expression of host microRNAs (miRs), non-coding RNAs that regulate gene expression, to promote survival of EBV+ B cell lymphomas. Here, we examined how the primary oncogene of EBV, latent membrane protein 1 (LMP1), regulates host miRs using an established model of inducible LMP1 signaling. LMP1 derived from the B95.8 lab strain or PTLD induced expression of the oncogene miR-155. However, PTLD variant LMP1 lost the ability to upregulate the tumor suppressor miR-193. Small molecule inhibitors (SMI) of p38 MAPK, NF-κB, and PI3K p110α inhibited upregulation of miR-155 by B95.8 LMP1; no individual SMI significantly reduced upregulation of miR-155 by PTLD variant LMP1. miR-155 was significantly elevated in EBV+ B cell lymphoma cell lines and associated exosomes and inversely correlated with expression of the miR-155 target FOXO3a in cell lines. Finally, LMP1 reduced expression of FOXO3a, which was rescued by a PI3K p110α SMI. Our data indicate that tumor variant LMP1 differentially regulates host B cell miR expression, suggesting viral genotype as an important consideration for the treatment of EBV+ B cell lymphomas. Notably, we demonstrate a novel mechanism in which LMP1 supports the regulation of miR-155 and its target FOXO3a in B cells through activation of PI3K p110α. This mechanism expands on the previously established mechanisms by which LMP1 regulates miR-155 and FOXO3a and may represent both rational therapeutic targets and biomarkers for EBV+ B cell lymphomas.

## Introduction

EBV is a ubiquitous γ-herpesvirus that has infected over 90% of the adult human population worldwide. Malignancies attributable to EBV represent 1.8% of global cancer deaths, with the burden increasing 14.6% from 1990 to 2010 (Khan and Hashim, 2014). When the immune system is compromised by post-transplant immunosuppression, primary immunodeficiency, HIV, immunomodulating biologicals, or advancing age, EBV can drive a spectrum of lymphoproliferations often involving B cells (Oyama et al., 2007; Asano et al., 2009; Hasserjian et al., 2009; Rizzi et al., 2009). For example, EBV+ post-transplant lymphoproliferative disorder (EBV+ PTLD) occurs in 1–20% of transplant recipients depending on the organ transplanted and results in significant morbidity and mortality (Martinez and Krams, 2017). The viral mechanisms that underlie the development of EBV+ B cell lymphomas such as PTLD are incompletely characterized but represent rational targets for therapeutic intervention.

EBV alters the expression of host genes including microRNA (miR), small non-coding RNA molecules that post-transcriptionally regulate gene expression by degrading or inhibiting the translation of target genes (Kaul and Krams, 2015). While the first virally encoded miRs were discovered in EBV (Pfeffer et al., 2004), the virus also alters host cell miR expression both in primary infection (Kaul et al., 2018) and in transformed cells (Mrázek et al., 2007; Cameron et al., 2008; Linnstaedt et al., 2010a,b; Forte et al., 2012; Rosato et al., 2012; Skalsky et al., 2012; Mansouri et al., 2014; Marquitz et al., 2014; Anastasiadou et al., 2015; Harris-Arnold et al., 2015). Viral genotype also plays a role in the specific miRs regulated by EBV (Harris-Arnold et al., 2015). For example, miR-194 is specifically downregulated in EBV+ PTLD and promotes B cell lymphoma survival by regulating IL-10 production (Harris-Arnold et al., 2015). In EBV-transformed B cells, miR-21, miR-34a, miR-146a, and miR-155 are among the most commonly upregulated host miRs (Mrázek et al., 2007; Cameron et al., 2008; Linnstaedt et al., 2010a; Forte et al., 2012; Rosato et al., 2012; Skalsky et al., 2012; Mansouri et al., 2014; Marquitz et al., 2014; Anastasiadou et al., 2015; Harris-Arnold et al., 2015).

The oncogenic miR-155 is a multi-functional miR with involvement in processes that range from the regulation of B cell function to muscle regeneration and tissue renewal (Mahesh and Biswas, 2019). A variety of miR-155 target genes have been verified including FOXO3a (*FOXO3*), PI3K p85α (*PIK3R1*), SHIP1 (*INPP5D*), PU.1 (*SPI1*), histone deacetylase 4 (*HDAC4*), and ubiquilin 1 (*UBQLN1*; Faraoni et al., 2009; O’Connell et al., 2009; Huang et al., 2012; Ling et al., 2013; Yang et al., 2015; Bayraktar and Roosbroeck, 2018). The context-dependent activity of miR-155 and its role as a regulator of diverse targets positions miR-155 as a highly influential component of cell fate and function. In EBV transformed B cells, miR-155 is a powerful oncogenic regulator, which is linked to activating the PI3K/Akt pathway, maintaining the viral genome copy number, and modulating the proliferation, growth, and survival of transformed cells (Lu et al., 2008; Linnstaedt et al., 2010a; Kim et al., 2012).

Four EBV genes can regulate miR-155 expression – the latent membrane proteins 1 and 2a (LMP1 and LMP2a) and the EBV nuclear antigens 2 and 3C (EBNA2 and EBNA3C; Gatto et al., 2008; Lu et al., 2008; Rahadiani et al., 2008; Yin et al., 2008, 2016; Du et al., 2011; Wang et al., 2011; Wood et al., 2018). These EBV latent genes co-operatively regulate miR-155 expression by activating host cell signaling pathways and transcription factors, such as NF-κB (Gatto et al., 2008; Rahadiani et al., 2008; Yin et al., 2016), p38 (Rahadiani et al., 2008), IRF4 (Wang et al., 2011; Wood et al., 2018), and AP-1 (Gatto et al., 2008; Rahadiani et al., 2008; Yin et al., 2008, 2016), by activating expression of other latent EBV genes (Wood et al., 2018) or by directly interacting with the regulatory regions of the miR-155 host gene (*miR-155HG*; Wood et al., 2018). For example, EBNA2 regulates expression of *miR-155HG* both directly, by binding an enhancer region upstream of the *miR-155HG* locus, and indirectly, by upregulating the expression of LMP1 and the transcription factor IRF4 (Wood et al., 2018). Upregulation of miR-155 by LMP1 can be blocked by small molecule inhibition of p38 and NF-κB and also requires NF-κB and AP-1 binding sites in the *miR-155HG* promoter (Gatto et al., 2008; Rahadiani et al., 2008). However, the precise mechanisms by which LMP1 regulates miR-155 and its targets in B cells remain to be determined.

LMP1 is the primary oncogene of EBV and is a functional, constitutively active mimic of the B cell costimulatory molecule CD40 (Uchida et al., 1999; Rastelli et al., 2008). LMP1 activates the p38 (Eliopoulos et al., 1999b; Vockerodt et al., 2001), Erk (Roberts and Cooper, 1998; Chuang et al., 2005), and JNK (Kieser et al., 1997; Eliopoulos et al., 1999a) mitogen-activated protein kinases, NF-κB (Huen et al., 1995; Eliopoulos et al., 1997), and PI3K/Akt (Dawson et al., 2003; Lambert and Martinez, 2007) host cell signaling pathways through its C-terminal activating regions 1 and 2 (CTAR1 and CTAR2). Activation of these pathways by LMP1 was primarily characterized using the B95.8 strain of EBV isolated from a patient with infectious mononucleosis (Skare et al., 1982). However, other naturally occurring LMP1 sequence variants have been isolated form EBV carriers, patients with EBV disease, and EBV-associated malignancies (Hu et al., 1991; Sandvej et al., 1997; Dawson et al., 2000; Fielding et al., 2001; Mainou and Raab-Traub, 2006; Guiretti et al., 2007; Vaysberg et al., 2008). Moreover, these LMP1 sequence variants display altered LMP1 function. For example, variants of LMP1 isolated from patients with EBV+ PTLD display sustained Erk activation and subsequent induction of c-Fos (Vaysberg et al., 2008). Whether tumor variants of LMP1 differentially regulate miR expression is unknown.

In this study, we compare how natural variants of LMP1, including those isolated from patients with EBV+ PTLD, regulate host B cell miRs. We provide evidence that tumor variant LMP1 differentially regulates host B cell miR expression and that each host B cell miR is regulated by a distinct subset of cell signaling pathways activated by LMP1. In the process, we uncover a novel mechanism by which LMP1 supports the expression of miR-155 and its target FOXO3a in host B cells.

## Materials and Methods

### Cell Lines

The EBV negative (EBV−) Burkitt’s lymphoma line BL41, which does not express endogenous LMP1, was kindly provided by Dr. Elliott Kieff (Harvard Medical School) and served as the parental cell line for our previously described lines expressing the following nerve growth factor receptor (NGFR).LMP1 constructs: B95.8, tumor variants 1–5, CTAR1mut, CTAR2mut, and the double mutant DMF3–2 (DM; Lambert and Martinez, 2007; Vaysberg et al., 2008). Tumor variant NGFR.LMP1 constructs were derived from the following EBV+ B cell lymphoma lines: 1 and 2 from AB5, 3 from JB7, 4 from MF4, and 5 from VB5. The following EBV positive (EBV+) B cell lymphoma lines were used: AB5, JB7, JC62, MF4, VB5, IMC-1, IMC-10, and IMC-34; these lines were generated as previously described (Beatty et al., 1997; Hatton et al., 2016). IMC-1, -10, and -34 are all B lymphoblastoid cell lines, while the others are spontaneously derived lymphoblastoid cell lines grown from peripheral blood or lymph nodes of patients diagnosed with EBV+ PTLD. The EBV− B cell lymphoma lines Pfeiffer and Toledo were also used; both are EBV− diffuse large B cell lymphoma (DLBCL) lines. Pfeiffer was acquired from American Type Culture Collection (ATCC), while Toledo was a gift from the Levy lab (Stanford University School of Medicine). All cell lines were cultured in complete RPMI (RPMI 1640 supplemented with 10% heat-inactivated fetal calf serum and 50 units/ml penicillin/streptomycin; ThermoFisher Scientific, Waltham, MA, USA) and maintained in a humidified, 37°C incubator with 5% CO_2_. Growth media for all cells expressing NGFR.LMP1 were supplemented with 700 μg/ml geneticin (Sigma-Aldrich, St. Louis, MO, USA; Lambert and Martinez, 2007).

### Activation of Latent Membrane Protein 1 Signaling by NGFR.LMP1 Crosslinking

After sub-culturing the day prior, NGFR.LMP1-expressing cells were resuspended in complete RPMI. LMP1 signaling was activated through the addition of unconjugated mouse anti-NGFR (0.5 μg/10^6^ cells, BioLegend, San Diego, CA, USA) for 30 min at room temperature followed by goat anti-mouse F(ab)_2_ (2 μg/10^6^ cells, Jackson ImmunoResearch, West Grove, PA, USA) for the indicated amount of time, as previously described (Lambert and Martinez, 2007). Expression and functionality of the NGFR.LMP1 construct were assayed by flow cytometry for NGFR and ICAM (CD54), respectively (Lambert and Martinez, 2007). Briefly, cells were washed with cold FACS buffer (PBS, 1% BSA, 0.02% sodium azide) and incubated on ice for 20 min with whole mouse IgG (Jackson ImmunoResearch). Cells were washed with FACS buffer and then incubated on ice with fluorescently conjugated primary antibodies. Stained cells were then washed and analyzed on a Millipore guava easyCyte5 (Colorado College) or a Miltenyi MACSQuant Analyzer 16 (Stanford University School of Medicine).

### Inhibition of Signal Transduction Pathways

The following small molecule inhibitors (SMI) were used to analyze the signaling pathways utilized by LMP1 to regulate host miRs: GDC-0941 [PI3K p110α, δ dual-specific inhibitor (Folkes et al., 2008), 1 μM], BYL719 [PI3K p110α inhibitor (Furet et al., 2013), varying concentrations as indicated], CAL-101 [PI3K p110δ inhibitor (Lannutti et al., 2011), 1 μM], Rapamycin (mTOR inhibitor, 10 ng/ml), PD98059 (Erk1,2 MAPK inhibitor, 10 μM), SB203580 [p38 MAPK inhibitor, which inhibits miR-155 regulation by LMP1 (Rahadiani et al., 2008), 5 μM], Bay11-7082 [an inhibitor of the ubiquitin system, which affects canonical NF-κB signaling (Strickson et al., 2013) and inhibits miR-155 regulation by LMP1 (Rahadiani et al., 2008), 5 μM], Cyclosporin A (500 ng/ml), and FK506 (10 ng/ml). All inhibitors were obtained from SelleckChem (Houston, TX, USA). Inhibition of these pathways and storage of inhibitors were as previously described (Lambert and Martinez, 2007; Hatton et al., 2011; Furukawa et al., 2013). The appropriate concentration of inhibitor was determined in preliminary experiments or was previously published and was based on the ability to specifically block phosphorylation of the relevant signaling molecule without inducing cell death or toxicity.

### Isolation of Exosomes and RNA and Quantitative Polymerase Chain Reaction for MicroRNAs

Exosomes were isolated using the ExoQuick-TC exosome isolation kit from System Biosciences (Palo Alto, CA, USA), per manufacturer’s instructions. Total RNA was isolated using the mirVana miRNA isolation kit form ThermoFisher Scientific, per manufacturer’s instructions. RNA was quantified using a NanoDrop ONE and stored at −80°C until use. Quantitative polymerase chain reaction (qPCR) was used to determine the relative expression of specific miRNA using TaqMan miRNA Assays [ThermoFisher Scientific, IDs 002623 (miR-155), 002367 (miR-193b), and 001223 (U47)]. Twenty or sixty nanogram of total RNA was used to generate target specific cDNA in single or multiplexed reactions, respectively, using the TaqMan miRNA Reverse Transcription Kit (ThermoFisher Scientific). After multiplexed reverse transcriptase reactions, cDNA was pre-amplified using the TaqMan PreAmp Master Mix (ThermoFisher Scientific). Target-specific amplification of miRs was assessed using TaqMan Universal Master Mix II, No AmpErase (ThermoFisher Scientific) on the BioRad CFX384 Touch Real-Time PCR Detection System (Stanford), or CFX Connect Real-Time PCR Detection System (Colorado College). The relative expression of each miRNA was calculated by first normalizing to the endogenous control U47 (Δ*C*_t_) and then to control samples as indicated (ΔΔ*C*_t_). For relative expression of miRNA, fold induction values (2^−ΔΔ*C*t^) are reported. For absolute quantification of miRNA, a standard curve was obtained using the Universal miRNA Reference (Agilent, Santa Clara, CA, USA).

### Western Blotting

Samples were collected and washed with PBS with 1 μM sodium orthovanadate. Lysates were generated as previously described (Hatton et al., 2011) or with 1× RIPA buffer (ThermoFisher Scientific) supplemented with 1× Halt Phosphatase and Protease Inhibitor (ThermoFisher Scientific) and 1 μM sodium orthovanadate (New England BioLabs, Ipswitch, MA, USA). Lysate concentration was determined using the Pierce 660 nm Protein Assay Kit (ThermoFisher Scientific) using a NanoDrop ONE. Samples were prepared to equal protein concentrations in 1× final Lamelli Sample Buffer (BioRad, Hercules, CA, USA) and stored at −20°C until use. Samples (20–40 μg/lane) were separated by SDS-PAGE and transferred to 0.2 μm nitrocellulose membranes. Membranes were blocked in 5% nonfat dry milk in 1× TBS/Tween and then probed with primary antibodies in either 5% nonfat dry milk or 5% BSA in 1× TBS/Tween. Primary antibodies specific for the following molecules were used: phosphorylated Akt (Ser473), HRP-conjugated Akt (pan), FOXO3a, PI3K p85α, SHIP1, and HRP-conjugated β-actin. All antibodies were from Cell Signaling Technologies (Danvers, MA, USA). When applicable, antibody binding was detected with HRP-conjugated secondary antibodies. Blots were developed with SuperSignal West Pico PLUS Chemiluminescent Substrate (ThermoFisher Scientific) according to the manufacturer’s instructions. Images were obtained on a ThermoFisher iBright FL1000 Imaging System (Colorado College) or an Azure Biosystems c300 Digital Imager (Stanford University School of Medicine). Densitometry was performed using ImageJ (National Institutes of Health).

## Results

### Tumor and Genetic Variants of Latent Membrane Protein 1 Differentially Regulate Host MicroRNAs

To determine which host miRs are regulated by LMP1, we utilized EBV− BL41 cells, which stably express chimeric NGFR.LMP1 molecules but not endogenous LMP1 (Lambert and Martinez, 2007; Vaysberg et al., 2008). In this model system, the addition of crosslinking anti-NGFR antibodies activates LMP1 signaling indistinguishable from full length LMP1 (Hatton et al., 2012). Using this inducible system, we compared miR expression before and after activation of LMP1 from the B95.8 lab strain of EBV. LMP1 activation induced upregulation of eight host B cell miR and downregulation of three host B cell miRs (Supplementary Table 1). We reasoned that those host miRs critical to LMP1 function, including maintaining latency, proliferation, and transformation, would be significantly and similarly regulated by both LMP1 and EBV (Harris-Arnold et al., 2015). No miRs were downregulated by both LMP1 and EBV. Three miRs, miR-146a, miR-155, and miR-193b, were upregulated by both LMP1 and EBV.

**Figure 1:**
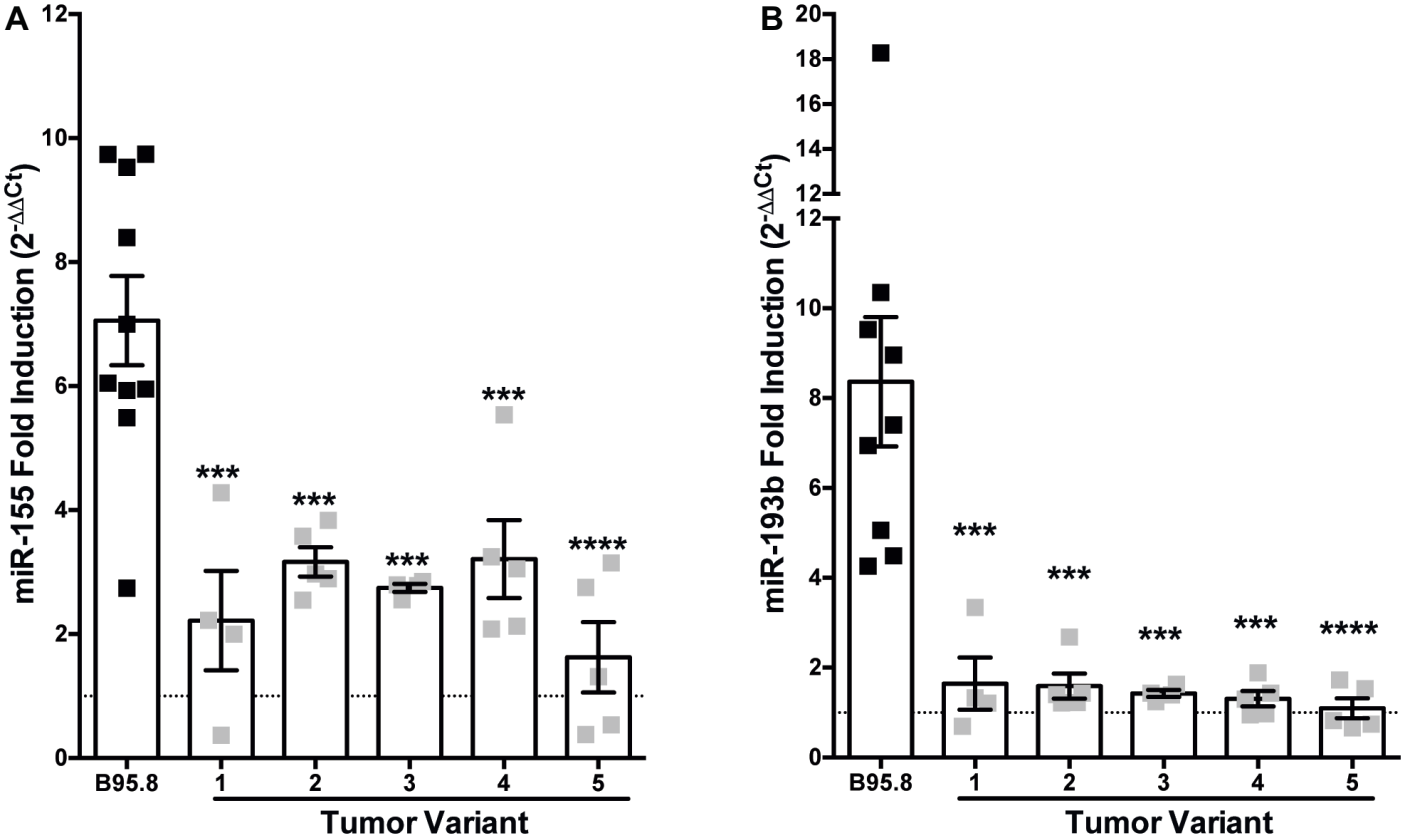
Tumor variants of LMP1 differentially regulate miR-155 and miR-193b from the B95.8 lab strain of LMP1. Two million EBV− BL41 cells expressing B95.8 lab strain or natural variant NGFR.LMP1 molecules were activated for 12 h prior to RNA isolation with the miRVana miRNA isolation kit. Relative expression of specific microRNAs was determined by quantitative PCR (qPCR) using TaqMan MicroRNA Assays. Target-specific cDNA was generated from 10 ng of total RNA using the TaqMan MicroRNA Reverse Transcription Kit and pre-amplified using the TaqMan PreAmp Master Mix. Finally, qPCR assays were performed using the TaqMan Universal Master Mix II, No AmpERASE UNG. The relative expression of **(A)** miR-155 and **(B)** miR-193b was calculated by first normalizing to the endogenous control U47 (ΔC_t_) and then to unactivated samples (ΔΔ*C*_t_). Fold-induction (2^−ΔΔ*C*t^) of each miR is shown. Each point represents an experimental replicate; two different lines expressing B95.8 NGFR.LMP1 were used. ^***^p ≤ 0.001, ^****^p ≤ 0.0001 by one-way ANOVA with *post hoc* multiple comparisons to activate B95.8 lab strain NGFR.LMP1.

While the B95.8 lab strain of EBV was originally isolated from a patient with infectious mononucleosis, other naturally occurring sequence variants of LMP1 have been isolated and demonstrated to display altered LMP1 function (Sandvej et al., 1997; Dawson et al., 2000; Fielding et al., 2001; Mainou and Raab-Traub, 2006; Vaysberg et al., 2008). We previously characterized and generated chimeric NGFR.LMP1 molecules of naturally occurring LMP1 tumor variants from EBV+ B cell lymphoma lines derived from patients with PTLD (EBV+ PTLD; Vaysberg et al., 2008). To determine whether these tumor variants of LMP1 differentially regulate host miRs, we examined the expression of miR-155 and miR-193b before and after LMP1 activation. Despite some variability of expression in B95.8 lab strain, there was no significant difference in the expression of NGFR.LMP1 between any of the lines tested (Supplementary Figure 1). Moreover, there was there was no significant correlation between expression of B95.8 NGFR.LMP1 and functionality, as measured by fold-change in ICAM expression after LMP1 activation (Supplementary Figure 1). miR-155 is induced an average of 2.6-fold by activation of tumor variant LMP1, which is significantly less than the 7.1-fold induction by B95.8 LMP1 (Figure 1A). While miR-193b is upregulated 8.4-fold by activated B95.8 LMP1, none of the tumor variant LMP1 regulates miR-193b (Figure 1B). These data demonstrate that variants of LMP1 isolated from EBV+ PTLD differentially regulate host miR-155 and miR-193b compared to B95.8 LMP1.

**Figure 2:**
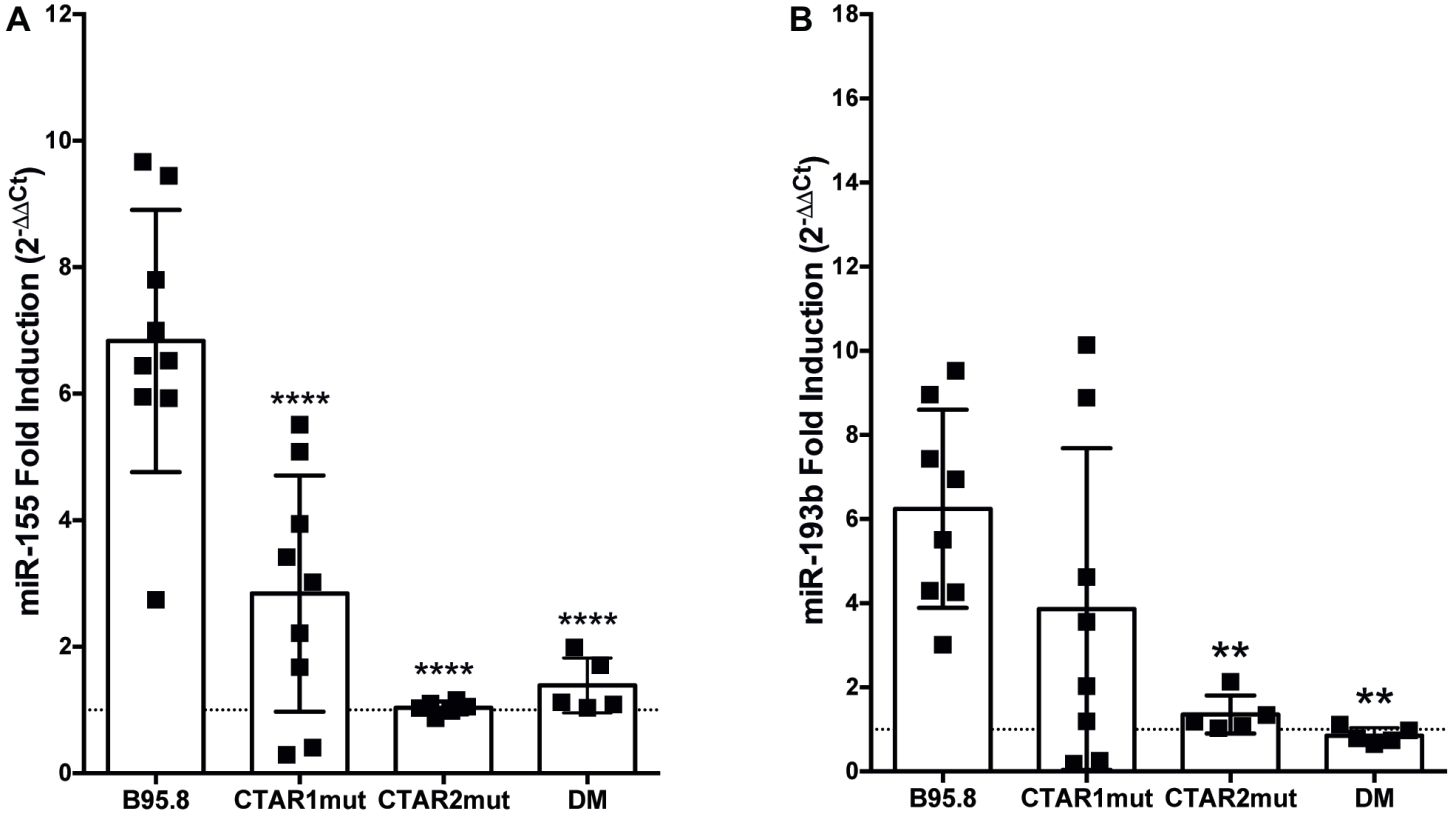
CTAR2 is essential for regulation of miRs by LMP1, while CTAR1 supports the regulation of miR-155, but not miR-193b. NGFR.LMP1 signaling was activated in 2 × 10^6^ EBV− BL41 cells expressing the following genetically engineered variants of the B95.8 NGFR.LMP1 molecules: wild type (B95.8), CTAR1 mutant (CTAR1mut), CTAR2 mutant (CTAR2mut), or containing both the G212S and S366T mutations commonly found in the natural variants of LMP1 (DM). After RNA isolation, relative expression of **(A)** miR-155b and **(B)** miR-193b was determined by quantitative PCR (qPCR) as described in Figure 1. Fold induction (2^−ΔΔCt^) of each miR is shown. Each point represents an experimental replicate; two different lines expressing B95.8 NGFR.LMP1 were used. ^**^p ≤ 0.01, ^****^p ≤ 0.0001 by one-way ANOVA with *post hoc* multiple comparisons to activate B95.8 lab strain NGFR.LMP1.

All of the LMP1 tumor variants, but not B95.8 LMP1, contain the same point mutations in their C-terminal domains, each within a specific CTAR: a glycine to serine mutation at amino acid 212 in CTAR1 and a serine to threonine mutation at amino acid 366 in CTAR2 (Vaysberg et al., 2008). To examine whether these tumor variant point mutations or CTAR regions are required for LMP1 to regulate miR-155 or miR-193b, we utilized chimeric B95.8 NGFR.LMP1 molecules modified to contain mutations in CTAR1 (CTAR1mut) or CTAR2 (CTAR2mut), or both tumor variant point mutations (DM; Lambert and Martinez, 2007; Vaysberg et al., 2008). CTAR2mut and DM fail to upregulate both miR-155 and miR-193b after LMP1 activation (Figure 2). DM NGFR.LMP1 expression was not significantly different from the other NGFR.LMP1 constructs (Supplementary Figure 1); therefore, the inability to regulate miR-155 and miR-193b is not due differences in NGFR.LMP1 expression. As we do not have data on CTAR1mut or CTAR2mut NGFR.LMP1 expression at the time of these experiments, it is possible that the inability of CTAR2mut to regulate both miR-155 and miR-193b is due to lack of NGFR.LMP1 expression. However, given that CTAR2 regulates the canonical NF-κB pathway (Paine et al., 1995; Saito et al., 2003; Gewurz et al., 2011), these data are consistent with previous reports demonstrating the involvement of NF-κB in regulation of miR-155 by LMP1 (Gatto et al., 2008; Rahadiani et al., 2008). Upregulation of miR-155 by B95.8 LMP1 is significantly reduced in the CTAR1mut (Figure 2A), but upregulation of miR-193b by B95.8 LMP1 is not significantly altered in the CTAR1mut (Figure 2B). Together, these data demonstrate that the CTAR2 and B95.8 variants at amino acids 212 and 366 are required to regulate both miR-155 and miR-193b. Moreover, CTAR1 supports the regulation of miR-155 by LMP1 but is dispensable for upregulation of miR-193b. Collectively, our data demonstrate that natural and genetic variants of LMP1 differentially regulate individual host miRs.

### Latent Membrane Protein 1 Utilizes Unique Host Signaling Pathways to Regulate Host MicroRNA Expression

**Figure 3:**
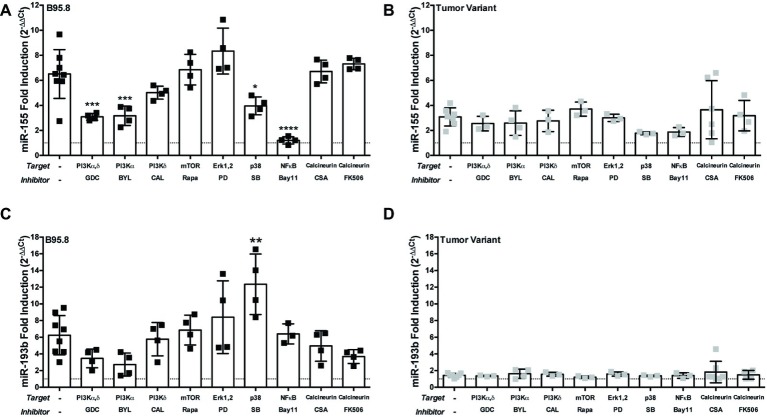
The PI3K p110α-specific inhibitor BYL719 inhibits maximal induction of miR-155 by LMP1 in B cells. Two million EBV− BL41 cells expressing B95.8 **(A,C)** or tumor variant #2 **(B,D)** NGFR.LMP1 molecules were activated in the presence of the indicated inhibitors for 12 h prior to RNA isolation with the miRVana miRNA isolation kit. Relative expression of specific microRNAs was determined by quantitative PCR (qPCR) as described in Figure 1. Fold induction (2^−ΔΔCt^) of each miR is shown. Each point represents an experimental replicate; two different lines expressing B95.8 NGFR.LMP1 were used. ^*^p ≤ 0.05, ^**^p ≤ 0.01, ^***^p ≤ 0.001, ^****^p ≤ 0.0001 by one-way ANOVA with *post hoc* multiple comparisons to activate NGFR.LMP1 in the absence of signaling inhibitors. Inhibitors used: GDC-0941 (GDC), BYL719 (BYL), CAL-101 (CAL), Rapamycin (Rapa), PD98059 (PD), SB203580 (SB), Bay11-7082 (Bay11), cyclosporin A (CSA), and FK506.

Each CTAR region in LMP1 is involved in activating specific host cell signaling pathways. For example, CTAR1 regulates the PI3K/Akt/mTOR pathway (Dawson et al., 2003; Mainou et al., 2005; Lambert and Martinez, 2007), while CTAR2 regulates the canonical NF-κB pathway (Paine et al., 1995; Saito et al., 2003; Gewurz et al., 2011). Thus, our results from the CTAR1mut, CTAR2mut, and DM suggest which signaling pathways may be critical in regulating expression of each host miR. To evaluate the requirement of specific host signal transduction pathways in the regulation of host miRs by LMP1, we activated LMP1 in the presence of small molecule inhibitors, which target members of the PI3K/Akt/mTOR, Erk, p38, NF-κB, or calcineurin pathways, and assayed for miR expression by qPCR. The small molecule inhibitors Bay11-7082, inhibitor of the ubiquitin system which affects canonical NF-κB signaling (Strickson et al., 2013), and SB203580, a p38 inhibitor, both reduce miR-155 induction by endogenous LMP1 (Rahadiani et al., 2008) and thus serve as positive controls. miR-155 upregulation by our chimeric, inducible LMP1 was ablated in the presence of Bay11-7082 and significantly reduced in the presence of SB203580 (Figure 3A). The requirement for canonical NF-κB signaling is consistent with our CTAR2mut data (Figure 2A). Thus, both NF-κB and p38 are required for regulation of miR-155 by LMP1 in our chimeric, inducible system. This is consistent with previous reports (Gatto et al., 2008; Rahadiani et al., 2008; Yin et al., 2008, 2016), validating our experimental approach.

Notably, miR-155 upregulation was significantly reduced by GDC-0941, a PI3K p110α,δ dual-specific inhibitor, and BYL719, a PI3K p110α-specific inhibitor, but not CAL-101, a PI3K p110δ-specific inhibitor (Figure 3A). BYL719 (alpesilib) has minimal effect on the other members of the class I catalytic PI3Ks p110β, p110γ, and p110δ (Furet et al., 2013) and was recently approved by the FDA for metastatic breast cancer. These data are consistent with our CTAR1mut data (Figure 2A), as the CTAR1 domain is required for activation of the PI3K pathway (Dawson et al., 2003; Mainou et al., 2005; Lambert and Martinez, 2007). Rapamycin, PD98059, cyclosporin A and FK506, inhibitors of mTOR, Erk, and calcineurin, respectively, had no significant effects on the upregulation of miR-155 by B95.8 LMP1 (Figure 3A). Individually, none of these small molecule inhibitors significantly altered the upregulation of miR-155 by tumor variant LMP1 (Figure 3B). miR-193b upregulation by B95.8 LMP1 was not significantly inhibited by any of the SMI (Figure 3C). However, B95.8 LMP1 activation in the presence of SB203580, a p38 inhibitor, leads to a significant increase in miR-193b compared to LMP1 activation alone (Figure 3C). Finally, activation of tumor variant LMP1 failed to upregulate miR-193b regardless of the presence of SMI (Figure 3D), consistent with our earlier findings (Figure 1B). Together, these data demonstrate that B95.8 LMP1 regulates miR-193b *via* p38, confirm the induction of miR-155 *via* NF-κB and p38, and uncover a novel supporting role for induction of miR-155 *via* PI3K p110α activation in B cells.

### PI3K p110α Is Required for MicroRNA-155 Upregulation by Latent Membrane Protein 1

**Figure 4:**
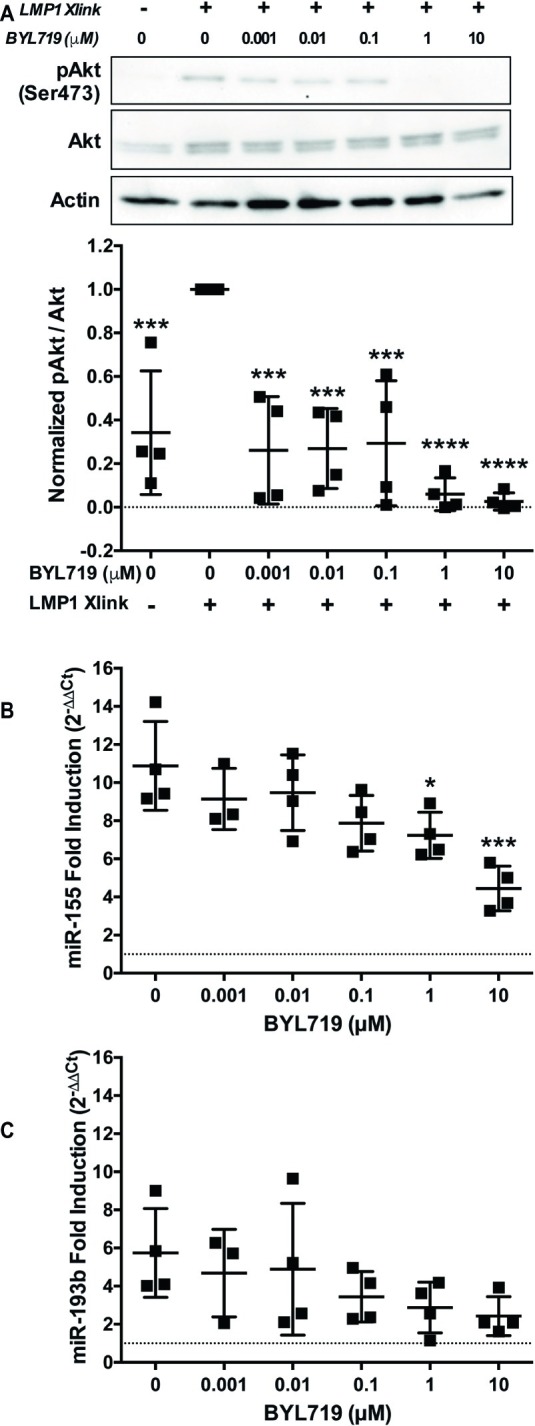
PI3K p110α is required for maximal induction of miR-155, but not miR-193b, by LMP1 in B cells. **(A–C)** Two million EBV− BL41 cells expressing B95.8 NGFR.LMP1 were activated in the presence of the indicated amounts of the PI3K p110α-specific inhibitor BYL719 for 12 h. **(A)** After lysate generation, Western blots for pAkt (Ser473), Akt, and actin were performed and imaged *via* the iBright FL1000 imaging system. Representative blots are shown. Densitometry was performed using ImageJ. All values shown were background subtracted, and the ratio of pAkt/Akt was first normalized to actin. Samples were then normalized to the sample where NGFR.LMP1 was activated in the absence of BYL719 to yield Normalized pAkt/Akt. Each point represents an experimental replicate. ^***^p ≤ 0.001, ^****^p ≤ 0.0001 by one-way ANOVA with *post hoc* multiple comparisons activated NGFR.LMP1 in the absence of signaling inhibitor; no other significant differences were observed. **(B,C)** After RNA isolation with the miRVana miRNA isolation kit, relative expression of **(B)** miR-155 and **(C)** miR-193b was determined by quantitative PCR (qPCR) as described in Figure 1. Fold induction (2^−ΔΔCt^) of each miR is shown. Each point represents an experimental replicate. ^*^p ≤ 0.05, ^***^p ≤ 0.001 by one-way ANOVA with *post hoc* multiple comparisons to activate NGFR.LMP1 in the absence of signaling inhibitor.

Inhibition of PI3K p110α by two different SMI, the PI3K p110α,δ dual-specific inhibitor GDC-0941 and the PI3K p110δ specific inhibitor BYL719, significantly reduced the upregulation of miR-155 by B95.8 LMP1 (Figure 3A). This strongly suggests that LMP1 upregulates miR-155 by activating PI3K p110α in B cells. To confirm activation of PI3K p110α by LMP1, we examined phosphorylation of Akt at serine 473 (pAkt^Ser473^), an indicator of PI3K p110α activity. B95.8 LMP1 activation induced pAkt^Ser473^ (Figure 4A, lanes 1 and 2). pAkt^Ser473^ induced by B95.8 LMP1 was most significantly and consistently reduced in the presence of 1 and 10 μM BYL719 (Figure 4A, lanes 6 and 7). These data demonstrate that LMP1 activates PI3K p110α and that BYL719 inhibits PI3K p110α function.

To further investigate whether LMP1 regulates miRs *via* PI3K p110α activation, we performed a titration of the PI3K p110α-specific inhibitor BYL719 and examined miR expression after LMP1 activation. miR-155 upregulation by B95.8 LMP1 significantly reduced by 1 and 10 μM BYL719 (Figure 4B). By contrast, miR-193b upregulation by B95.8 LMP1 was not significantly reduced by any concentration of BYL719 (Figure 4C). Given that BYL719 inhibits PI3K p110α function in our system at 1 and 10 μM (Figure 4A), our data suggest that LMP1 activates PI3K p110α to regulate miR-155, but not miR-193b, expression in B cells.

### Epstein-Barr Virus Reduces Expression of the MicroRNA-155 Target FOXO3a

**Figure 5:**
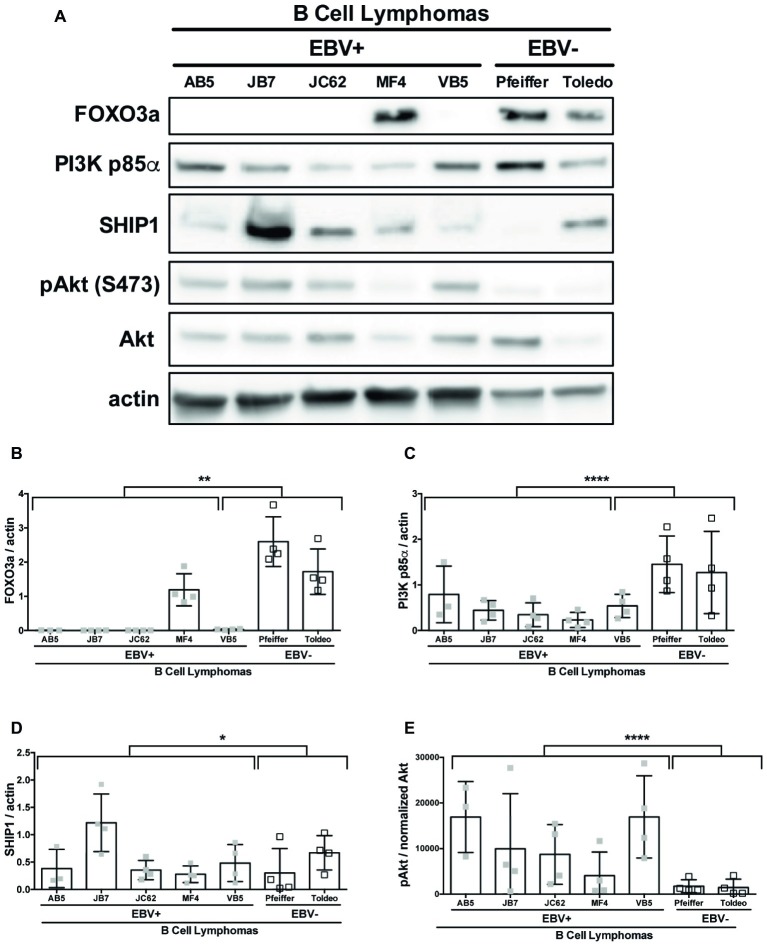
miR-155 targets FOXO3a and PI3K p85α are downregulated in EBV+ B cell lymphomas. Lysates from EBV+ and EBV− B cell lymphomas were generated with RIPA buffer supplemented with 1× Halt Protease and Phosphatase Inhibitors and 1 mM sodium orthovanadate and quantified using the Pierce 660 nm Protein Assay. About 30 μg of protein was loaded on a 4–20% tris-glycine gel and subsequently transferred to a nitrocellulose membrane. Western blots for the indicated proteins were performed as per manufacturer’s instructions and imaged *via* the Azure c300 digital imager. Representative blots are shown in **(A)**. Densitometry was performed using ImageJ, and all values were background subtracted and normalized to the indicated loading control **(B–E)**. Each point represents an experimental replicate. ^*^p ≤ 0.05, ^**^p ≤ 0.01, ^****^p ≤ 0.0001 by linear mixed effects model.

SHIP1, PI3K p85α, and FOXO3a are all known targets of miR-155 (O’Connell et al., 2009; Huang et al., 2012; Ling et al., 2013). To determine if these targets of miR-155 were regulated by EBV, we compared the constitutive expression of these proteins in EBV+ and EBV− B cell lymphomas by Western blot (Figure 5A). FOXO3a expression was significantly decreased in EBV+ B cell lymphoma lines compared to EBV− B cell lymphomas lines (Figures 5A,B). Notably, FOXO3a expression was not detectable in four of the five EBV+ B cell lymphoma lines we examined. Similarly, while all EBV+ B cell lymphoma lines express PI3K p85α, its expression was also significantly decreased in these lines compared to EBV− B cell lymphoma lines (Figures 5A,C). While there was a significant difference in expression of SHIP1 between EBV+ and EBV− B cell lymphoma lines, this is driven by the strong expression of SHIP1 in the EBV+ B cell lymphoma line JB7 (Figures 5A,D). Together our data suggest that EBV decreases expression of the miR-155 targets FOXO3a and PI3K p85α.

Both SHIP1 and PI3K p85α are negative regulators of the PI3K pathway (Huang et al., 2012). To determine if expression of these miR-155 targets correlates with the status of PI3K pathway activity, we examined pAkt^Ser473^ in EBV+ and EBV− B cell lymphoma cell lines. EBV+ B cell lymphoma lines display robust pAkt^Ser473^ compared to EBV− B cell lymphoma lines (Figures 5A,E). Thus, EBV-transformed cells display constitutive PI3K pathway activation and reduced PI3K p85α expression. Together, our data suggest that PI3K pathway activation correlates with EBV status and is inversely correlated with PI3K p85α expression.

**Figure 6:**
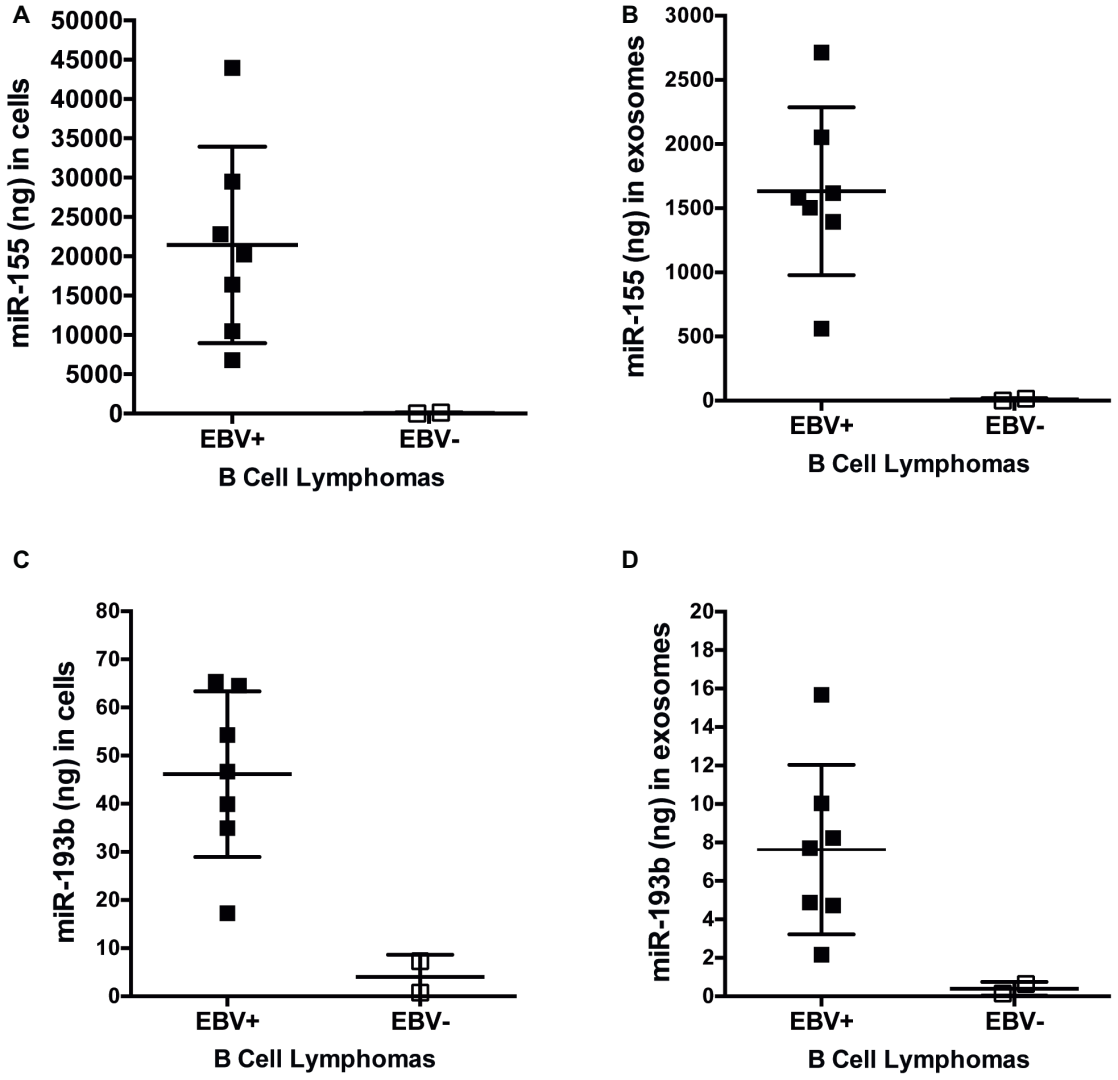
miR-155 and miR-193b are increased in EBV+ B cell lymphomas and their associated exosomes. Exosomes were isolated from the supernatants of EBV+ and EBV− B cell lymphoma using the ExoQuick-TC exosome isolation kit. RNA from cells **(A,C)** and exosomes **(B,D)** was isolated with the miRVana miRNA isolation kit. Expression of specific microRNAs was determined by quantitative PCR (qPCR) as described in Figure 1. For absolute quantification of these miRs, a standard curve was also performed using Universal Human miRNA Reference RNA. Each point represents unique EBV+ or EBV− B lymphoma cell line.

miRs function to regulate gene expression by binding to the 3′-UTR of target mRNA molecules, which, in most cases, mark them for degradation by the RNA-induced silencing complex (RISC), resulting in an inverse correlation with expression of a given miR and its targets. To determine if expression of miR-155 targets inversely correlates with miR-155 expression, we quantified the expression of miR-155 and miR-193b in EBV+ and EBV− B cell lymphoma cell lines and their associated exosomes by qPCR. On average, 21,450 ± 4,720 and 1,632 ± 247 ng of miR-155 are produced by EBV+ B cell lymphomas and associated exosomes, respectively, which are significantly greater than the 63.9 ± 57.3 and 8.5 ± 7.6 ng produced by EBV− B cell lymphomas and associated exosomes (Figures 6A,B). Similarly, an average of 46.2 ± 6.5 and 7.6 ± 1.7 ng of miR-193b is produced by EBV+ B cell lymphomas and associated exosomes, respectively, while only 4 ± 3.3 and 0.4 ± 0.2 ng are produced by EBV− B cells and their associated exosomes (Figures 6C,D). Together, these data demonstrate that miR-155 and miR-193b are elevated in EBV+ B cell lymphomas and their associated exosomes. While exosomal miR content is comparatively reduced, it mirrors cellular miR content. Moreover, these data demonstrate that miR-155 levels inversely correlate with the expression of the miR-155 targets FOXO3a and PI3K p85α with high miR-155 levels and reduced FOXO3a and PI3K p85α levels in EBV+ B cells lymphomas.

### Latent Membrane Protein 1 Regulates Expression of the MicroRNA-155 Target FOXO3a *via* PI3K p110α

**Figure 7:**
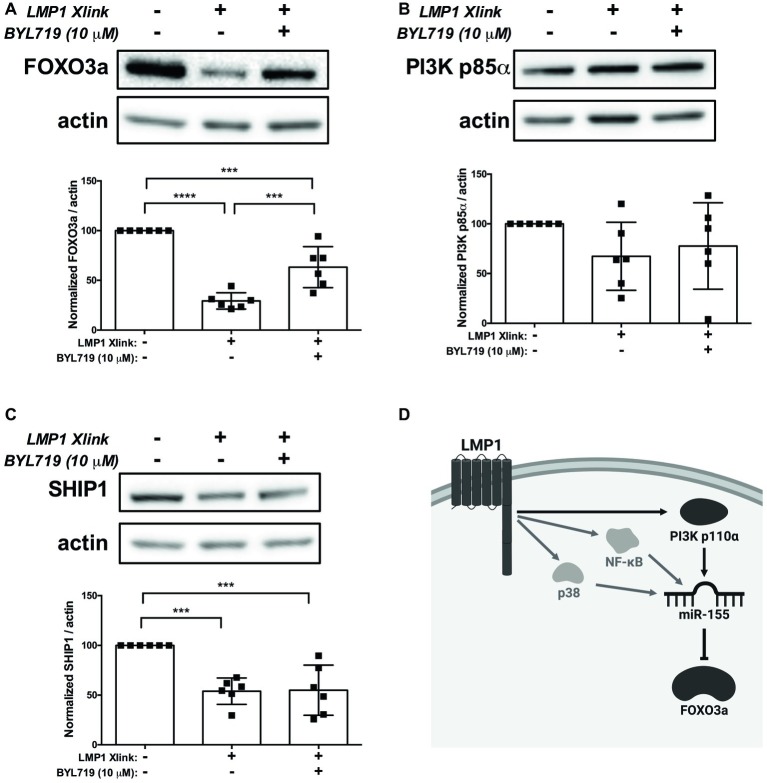
LMP1 downregulates expression of the miR-155 target FOXO3a *via* PI3K p110α in B cells. **(A–C)** Four million EBV- BL41 cells expressing B95.8 NGFR.LMP1 were treated as indicated for 16 h prior to lysis. Lysates were generated using phospholysis buffer supplemented with 1× Halt Protease and Phosphatase Inhibitors and 1 mM sodium orthovanadate and quantified using the Pierce 660 nm Protein Assay. Lysates were loaded on a 4–20% tris-glycine gel and subsequently transferred to a nitrocellulose membrane. Western blots for the indicated proteins were performed as per manufacturer’s instructions and imaged *via* the iBright FL1000 imaging system. Representative blots are shown. Densitometry was performed using ImageJ, and all values shown were background subtracted and normalized to actin. Each point represents an experimental replicate. ^***^p ≤ 0.001, ^****^p ≤ 0.0001 by one-way ANOVA with *post hoc* multiple comparisons. **(D)** Proposed model by which B95.8 LMP1 supports the regulation of miR-155 and FOXO3a through PI3K p110α in B cells (dark gray). As shown, our data also confirm previous findings regarding the involvement of NF-κB and p38 in the upregulation of miR-155 by LMP1 (light gray). Created with BioRender.com.

Collectively, our data expand on the previously established mechanisms of miR-155 regulation by LMP1 and suggest a model, wherein EBV, *via* activation of PI3K p110α by LMP1, regulates expression of miR-155 and its target FOXO3a in B cells. To test our model, we activated LMP1 in the absence and presence of the PI3K p110α-specific inhibitor BYL719 and examined expression of the miR-155 targets FOXO3a, PI3K p85α, and SHIP1 by Western blot. B95.8 LMP1 activation significantly decreased FOXO3a expression compared to basal levels and BYL719 partially rescued FOXO3a expression (Figure 7A), as predicted by this model. Tumor variant LMP1 activation also significantly decreased FOXO3a expression compared to basal levels (Supplementary Figure 2). However, BYL719 treatment did not rescue FOXO3a expression after activation of tumor variant LMP1 activation (Supplementary Figure 2), consistent with our earlier findings (Figure 3B). B95.8 LMP1 activation, regardless of BYL719 treatment, did not significantly alter PI3K p85α expression (Figure 7B). Finally, while B95.8 LMP1 activation significantly reduced SHIP1 expression, BYL719 treatment did not rescue SHIP1 expression (Figure 7C). Together, our data suggest that both B95.8 and tumor variant LMP1 regulate expression of the miR-155 target FOXO3a in B cells. Moreover, B95.8 LMP1 regulates FOXO3a *via* PI3K p110α activation.

## Discussion

The viral mechanisms underlying the development and progression of EBV+ B cell lymphomas provide rational, virally controlled targets for the treatment of these malignancies. We previously demonstrated that transformed B cells from patients with EBV+ PTLD display miR profiles that are distinct from B cells transformed with B95.8 EBV (Harris-Arnold et al., 2015). Moreover, these tumor variants of EBV uniquely alter expression of miRs, such as miR-194, to drive the survival of EBV+ B cell lymphomas (Harris-Arnold et al., 2015). How natural variants of the primary oncogene LMP1 contribute to these unique profiles had not been examined. Using an established chimeric, inducible model of LMP1 signaling (Lambert and Martinez, 2007; Vaysberg et al., 2008; Hatton et al., 2012), we provide evidence that tumor and genetic variants of LMP1 differentially regulate host miR-155 and miR-193b. Moreover, we demonstrate that LMP1 utilizes distinct host signaling pathways to regulate host miR expression; specifically, miR-193b is regulated by p38, while miR-155 is regulated by NF-κB, p38, and PI3K p110α. Notably, we provide the first evidence that the activation of PI3K p110α by LMP1 supports the regulation of miR-155 and its target FOXO3a in B cells (Figure 7D).

Tumor variant LMP1 from EBV+ PTLD displays altered host B cell miR expression compared to B95.8 LMP1. Specifically, tumor variant LMP1 exhibited reduced miR-155 expression and lack of the ability to regulate miR-193b expression compared to B95.8 LMP1. Moreover, B95.8 LMP1 molecules containing the two tumor variant point mutations, G212S and S366T, display reduced or lost ability to upregulate miR-155 and miR-193b, respectively. The consequence of reduced miR-155 expression by tumor variant LMP1 remains to be fully determined. However, reduced miR-155 induction did not alter the ability of tumor variant LMP1 to significantly reduce expression of the miR-155 target FOXO3a. Notably, miR-193b functions as a tumor suppressor in melanoma, pancreatic cancer, breast cancer, and prostate cancer by suppressing targets including uPA, Mcl1, and cyclin D1 (Li et al., 2009; Chen et al., 2010, 2011; Rauhala et al., 2010; Jian et al., 2014; Kaukoniemi et al., 2015). Thus, it is of future interest to examine the functional consequences of tumor variant LMP1’s inability to regulate miR-193b. Collectively, our data provide additional evidence that host B cell miR expression is altered by EBV, specifically by the primary oncogene LMP1, in the progression to EBV+ B cell lymphomas (Mrázek et al., 2007; Cameron et al., 2008; Linnstaedt et al., 2010b; Forte et al., 2012; Rosato et al., 2012; Skalsky et al., 2012; Harris-Arnold et al., 2015). Moreover, our data suggest that viral genotype is critical in the development of EBV+ B cell lymphomas, as sequence variants that dysregulate miRs, such as miR-193b, may have a selective growth advantage that drives lymphomagenesis. Looking forward, an unbiased approach examining which host miRs are regulated by tumor variant LMP1 molecules may help determine if tumor variant LMP1 gains the ability to regulate any host miRs that are not regulated by B95.8 LMP1 and thus may drive lymphomagenesis.

Our data indicate that each host B cell miR is regulated by a distinct subset of cell signaling pathways activated by LMP1, with NF-κB, p38, and PI3K p110α required for miR-155 upregulation and p38 required for miR-193b inhibition by B95.8 LMP1. Additionally, this suggests that there is no global dysregulation of miR expression or biogenesis by LMP1. As our study focused on examining each pathway in isolation, it will be critical to examine the mechanisms by which these pathways coordinate to regulate miR-155 and miR-193b expression. LMP1 also activates the JNK pathway (Eliopoulos et al., 1999b; Vockerodt et al., 2001). While inhibition of the JNK pathway with the small molecule inhibitor SP600126 did not alter the regulation of *miR-155HG* in B cells (Rahadiani et al., 2008), it will also be of interest to study if JNK cooperates with the NF-κB, p38, and PI3K pathways to regulate miR-155. Moreover, we demonstrate that no individual pathway activated by tumor variant LMP1 is sufficient for regulation of miR-155. While these tumor variants share the common mutations at positions 212 and 366, the only defined alterations to signaling are sustained Erk activation and subsequent activation of c-Fos (Vaysberg et al., 2008). However, Erk is dispensable for upregulation of miR-155 by both B95.8 and tumor variant LMP1. Collectively, this suggests that the variations in tumor variant LMP1, specifically the point mutations at positions 212 and 366, alter the cooperativity between signaling pathways, result in the activation of novel signaling pathways, or lose the regulation of another signaling pathway activated by B95.8 LMP1. Along with an unbiased approach to examine the host miRs regulated by tumor variant LMP1, it will be critical to define how the point mutations at positions 212 and 366 affect the NF-κB, p38, PI3K, and JNK signaling pathways to gain a more thorough picture of how tumor variant LMP1 alters the landscape of host signaling and miR expression.

Our data add to the larger, established mechanisms of miR-155 regulation by EBV by providing the first direct evidence that activation of the p110α catalytic isoform of PI3K by LMP1 supports the regulation of mature miR-155 in B cells. Previously, Rahadiani et al. used the small molecule inhibitor LY294002 to conclude that this pathway was not involved in regulation of miR-155 by LMP1 in B cells. However, the limitations of LY294002 have been more widely noted since this publication, and include its weak potency and significant off-target effects on other kinases (Workman et al., 2010). Our approach focused on using multiple new and/or more selective inhibitors for the PI3K p110α and p110δ catalytic isoforms and mTOR, as well as genetic mutants of the CTAR1 region required for PI3K pathway activation by LMP1 (Dawson et al., 2003; Mainou et al., 2005; Lambert and Martinez, 2007). While we cannot formally exclude the possibility of off-target effects of these small molecule inhibitors, the PI3K kinase pathway has previously been indirectly implicated in regulation of miR-155 by LMP1. Notably, both Src and PI3K are required for activation of IRF4 by LMP1 (Wang et al., 2016), and IRF4 is required to regulate expression of the primary transcript of miR-155, *miR-155HG* or *BIC*, in EBV-transformed cells (Wang et al., 2011; Wood et al., 2018). Finally, LMP1 is one of four EBV genes, including LMP2a, EBNA2, and EBNA3C, which have been demonstrated to regulate miR-155 expression (Gatto et al., 2008; Lu et al., 2008; Rahadiani et al., 2008; Yin et al., 2008, 2016; Du et al., 2011; Wang et al., 2011; Wood et al., 2018). Collectively, our data and that of Wang et al. suggest additions to the model proposed by Wood et al., where EBNA2A directly regulates *miR-155HG* expression and indirectly regulates *miR-155HG* expression through the upregulation of both LMP1 and IRF4 (Wood et al., 2018). In this updated model, LMP1 can then activate or support miR-155 expression in the following ways: by PI3K p110α activation of IRF4 (Wang et al., 2011; Wood et al., 2018), by NF-κB activation (Gatto et al., 2008; Rahadiani et al., 2008; Yin et al., 2016), by p38 activation (Rahadiani et al., 2008), and by AP-1 activation (Gatto et al., 2008; Rahadiani et al., 2008; Yin et al., 2008, 2016). As LMP2a also activates similar signaling pathways to LMP1, including the PI3K pathway, further examination of how these four EBV genes cooperate with each other and host signaling pathways to regulate miR-155 expression is warranted.

In addition, we provide the first evidence that activation of PI3K, specifically the p110α isoform, by LMP1 regulates the miR-155 target FOXO3a in B cells. Supporting this, we observe an inverse correlation between FOXO3a expression in cell lines and miR-155 levels in both B cells and their associated exosomes based on EBV status. All of the EBV+ B cell lines examined express LMP1, with some variability in expression across lines (Vaysberg et al., 2009; Hatton et al., 2012). It is possible that other, EBV-independent, mechanisms exist through which FOXO3a expression or activity is regulated in B cell lymphomas. FOXO3a is regulated not only post-transcriptionally by miRs, but also by post-translational modifications and protein-protein interactions, which alter its subcellular location and half-life (Liu et al., 2018; Fasano et al., 2019). For example, Akt, Erk, serum-and glucocorticoid-inducible kinases (SGK), and IκB kinase isoform β (IKKβ) can all promote the nuclear export of FOXO3a. Once in the cytoplasm, FOXO3a is ubiquitinated and targeted for degradation by the proteasome (Yang et al., 2008). Collectively, however, our data strongly suggest that LMP1 post-transcriptionally regulates FOXO3a expression in B cells by activating the PI3K p110α isoform to induce the expression of miR-155. This may be a complementary mechanism to the regulation of FOXO3a by LMP1 observed in epithelial cells, where activation of Akt by CTAR1 of LMP1 induces phosphorylation and nuclear export of FOXO3a (Chen et al., 2008; Lo et al., 2010). The existence of multiple mechanisms by which LMP1 can regulate FOXO3a function strongly suggests its importance in transformation by EBV. A member of the forkhead transcription factor family, FOXO3a, regulates expression of key genes involved in apoptosis, proliferation, survival, autophagy, and DNA repair (Liu et al., 2018). Notably, FOXO3a functions as a tumor suppressor in breast cancer (Hu et al., 2004; Zou et al., 2008) and natural killer cell neoplasms (Karube et al., 2011), and low FOXO3a expression is associated with poor prognosis in glioma (Shi et al., 2010), ovarian cancer (Fei et al., 2009), and nasopharyngeal carcinoma (Shou et al., 2012). Thus, the downregulation of FOXO3a expression by EBV *via* LMP1 may serve as a mechanism by which EBV establishes or maintains transformation of human B cells.

While FOXO3a is regulated by the PI3K pathway, both SHIP1 and PI3K p85α are negative regulators of the PI3K pathway^23,24^. We observe an inverse correlation between PI3K p85α expression and pAkt^Ser473^, where there is reduced PI3K p85α expression and increased pAkt^Ser473^ in EBV-transformed B cells. Moreover, we similarly observe an inverse correlation between PI3K p85α expression and miR-155 levels in both B cells and their associated exosomes based on EBV status. These correlations suggest a positive feedback loop between activation of the PI3K pathway by LMP1 to induce miR-155 expression and regulation of PI3K pathway inhibitors such as PI3K p85α by miR-155 (Huang et al., 2012). While we observe significant downregulation of PI3K p85α expression by EBV, there is no significant change upon LMP1 activation. This suggests that PI3K p85α regulation by LMP1 may involve different kinetics or cooperation with other latent viral genes to achieve reduced PI3K p85α expression. SHIP1 is significantly downregulated by LMP1, but not by EBV. This suggests that other latent viral genes may function to induce SHIP1 expression.

In preclinical models, we have previously demonstrated that PI3K pathway inhibition is a rational therapeutic target for EBV+ PTLD (Furukawa et al., 2013; Hatton et al., 2013; Sang et al., 2019); our data here suggest that one mechanism by which this works is by reducing miR-155 expression. miR-155 inhibitors, such as MRG-106 (miRagen Therapeutics Inc.), are currently in phase I clinical trials for cutaneous T cell lymphoma, chronic lymphocytic leukemia, diffuse large B cell lymphoma, and adult T cell leukemia/lymphoma (ClinicalTrials.gov Identifier NCT02580552). Gene therapy to restore FOXO3a expression has been pursued in oral squamous cell carcinoma (Fang et al., 2011). FOXO3a expression and cellular or exosomal miR-155 may be suitable biomarkers for EBV+ PTLD. Finally, viral genotype is an important consideration for the treatment of EBV+ PTLD, as we observe different requirements for PI3K p110α activation by B95.8 and tumor variant LMP1 in the regulation of miR-155 and FOXO3a. Together, our results suggest that understanding the regulation and function of miRs can identify potential therapeutic targets for EBV+ B cell lymphomas such as PTLD.

## Data Availability Statement

The dataset generated for this study can be found in the Gene Expression Omnibus (GEO) *via* accession number GSE136721.

## Author Contributions

OH, AH-A, SK, and OM conceived and designed the study. OH, MMS, MA, MM, CS, MWS, SH, GD, JM, KR, AH-A, and EM collected the data. OH, MMS, MA, MM, CS, MWS, SH, OM, and SK analyzed the data. OH, MMS, MA, MM, CS, MWS, SK, and OM prepared the manuscript.

### Conflict of Interest

The authors declare that the research was conducted in the absence of any commercial or financial relationships that could be construed as a potential conflict of interest.
