# Supplementary material for: Epstein-Barr Virus Latent Membrane Protein 1 Regulates Host B Cell MicroRNA-155 and Its Target FOXO3a via PI3K p110α Activation
Source: Front Microbiol. 2019 Nov 26;10:2692. doi: 10.3389/fmicb.2019.02692 (PMC6988802; doi:10.3389/fmicb.2019.02692)
Supplement: Supplementary file 1 [file Data_Sheet_1.docx]

Supplementary Material

# Supplementary Method – microRNA (miR) Array

To determine expression of 377 human miRs, the TaqMan Array Human miRNA Card A v3.0 (Life Technology) was run on the ABI 7900HT System (Stanford Functional Genomics Facility). The array data was analyzed as previously described for EBV transformed cells (Harris‐Arnold et al., 2015). Briefly, Ct values were first normalized to the median Ct value of the individual array (ΔCt), The ΔCt of the miRNA in the activated LMP1 samples (n=2) was then normalized to the ΔCt from the matched unactivated LMP1 samples (n=2, ΔΔCt). Fold change was calculated as 2^-ΔΔCt^.

# Supplementary Table 1

| **Supplemental Table 1. miRs Regulated by LMP1 Activation*** | |
| --- | --- |
| **Upregulated** | **Downregulated** |
| miR-146a** | miR-218 |
| miR-155** | miR-338-3p |
| miR-193b** | miR-618 |
| miR-222 |  |
| miR-449b |  |
| miR-503 |  |
| miR-520b |  |
| miR-548b-5p |  |
| **= greater or less than 2-fold upon LMP1 activation in both of the n=2 replicates*  *** = similarly regulated by B95.8 EBV, Harris-Arnold et al, AJT, 2015* | |

# Supplementary Figures


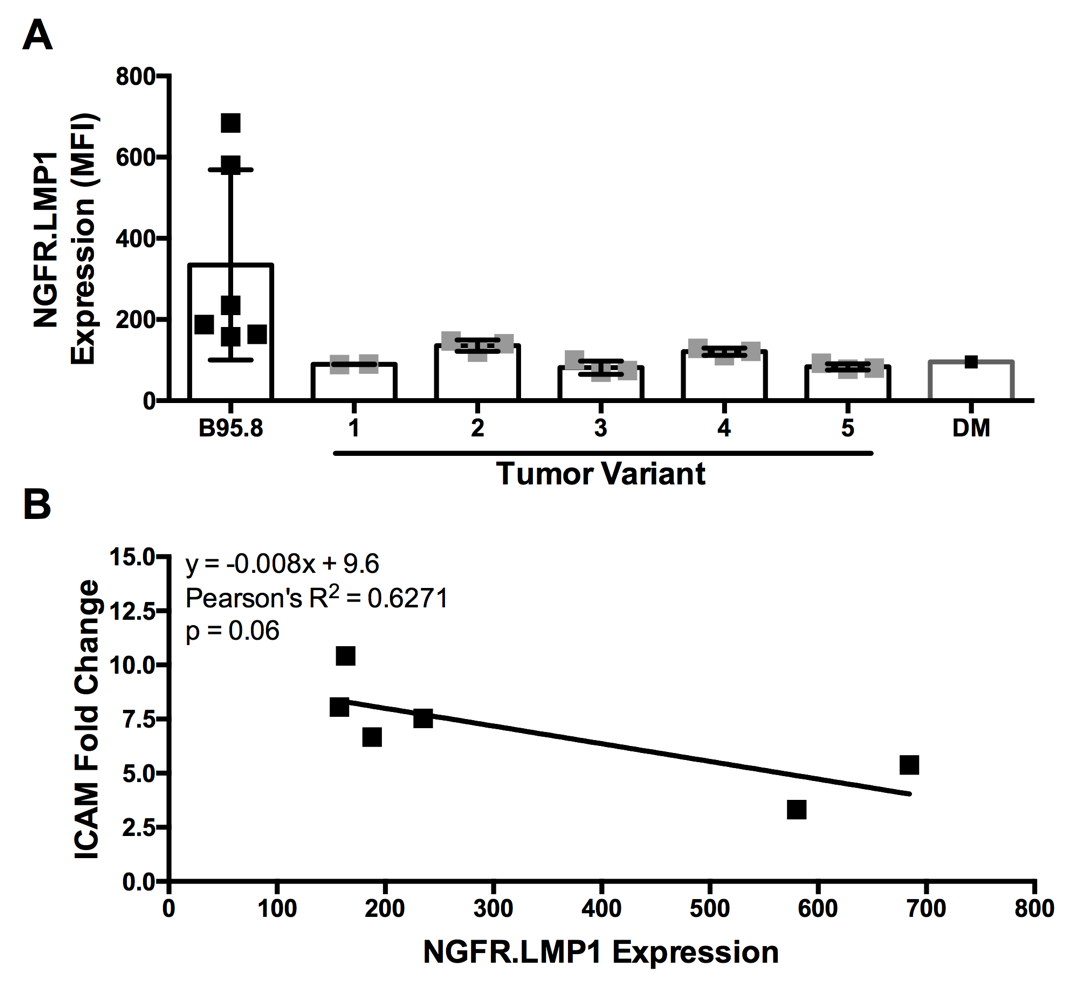


**Supplementary Figure 1. NGFR.LMP1 Expression and Correlation with Functionality.** One million EBV- BL41 cells containing the indicated NGFR.LMP1 chimeric construct were activated overnight. Cells were then collected and washed with 1X FACS buffer. After incubation with a mouse IgG blocking antibody, cells were stained with NGFR-PE (A) or ICAM-PE and collected on a MACSQuant. Median fluorescence intensity (MFI) was calculated on forward- and side-scatter gated cells. Fold change in ICAM expression was calculated by dividing the MFI of ICAM-PE on activated cells by unactivated cells. Each point represents an experimental replicate. * p ≤ 0.05 by one-way ANOVA with post-hoc multiple comparisons. (B) Correlation analysis of B95.8 NGFR.LMP1 expression and fold-change in ICAM, a measure of NGFR.LMP1 functionality. There is no significant correlation between NGFR.LMP1 expression and functionality.


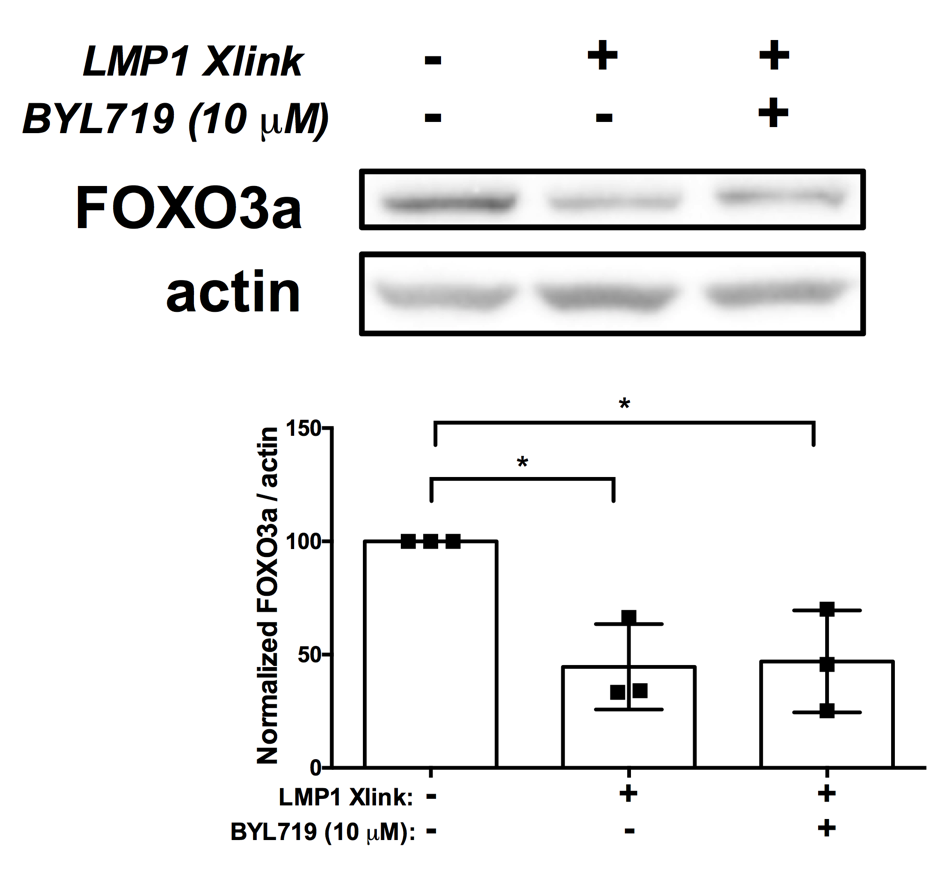


**Supplementary Figure 2. Tumor Variant LMP1 downregulates expression of the miR-155 target FOXO3a independently of PI3K p110α.** Four million EBV- BL41 cells expressing tumor variant #2 NGFR.LMP1 were treated as indicated for 16 hours prior to lysis. Lysates were generated using phospholysis buffer supplemented with 1X Halt Protease and Phosphatase Inhibitors and 1 mM sodium orthovanadate and quantified using the Pierce 660nm Protein Assay. Lysates were loaded on a 4-20% tris-glycine gel and subsequently transferred to a nitrocellulose membrane. Western blots for the indicated proteins were performed as per manufacturer’s instructions and imaged via the iBright FL1000 imaging system. Representative blots are shown. Densitometry was performed using ImageJ and all values shown were background subtracted and normalized to actin. Each point represents an experimental replicate. * p ≤ 0.05 by one-way ANOVA with post-hoc multiple comparisons.
